# Supplementary material for: Berbamine Reduces Chloroquine-Induced Itch in Mice through Inhibition of MrgprX1
Source: Int J Mol Sci. 2022 Nov 18;23(22):14321. doi: 10.3390/ijms232214321 (PMC9698483; doi:10.3390/ijms232214321)
Supplement: Supplementary file 1 [file ijms-23-14321-s001.zip › ijms-1997952-supplementary.pdf]

Supporting information

Berbamine reduces chloroquine-induced itch in mice through inhibition of MrgprX1

Kunhi Ryu, Yunkyung Heo, Yechan Lee, Hyejin Jeon and Wan Namkung *

[1]College of Pharmacy, Yonsei Institute of Pharmaceutical Sciences, Yonsei University, Incheon 21983, Korea.

Correspondence to: Wan Namkung, Ph.D. College of Pharmacy, Yonsei Institute of Pharmaceutical Sciences, Yonsei University, Incheon 21983, Korea; Tel: +82 32 749 4519; Fax: +82 32 749 4109; E-mail: wnamkung@yonsei.ac.kr

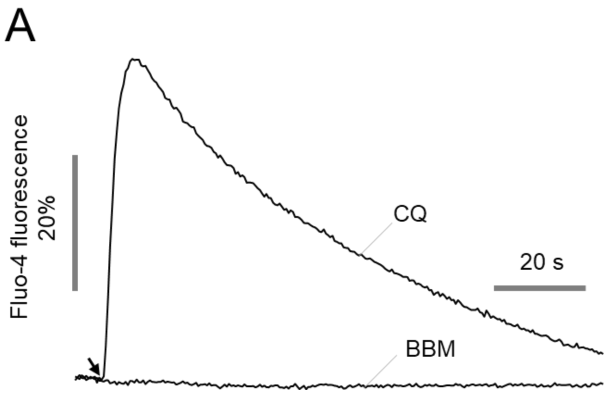

Figure S1. Effect of berbamine on MrgprX1 activation. (**A**) Intracellular calcium levels were measured using Fluo-4 NW in HEK293T cells expressing MrgprX1. The cells were treated with 30 μM berbamine (BBM) or 1 mM of chloroquine (CQ).

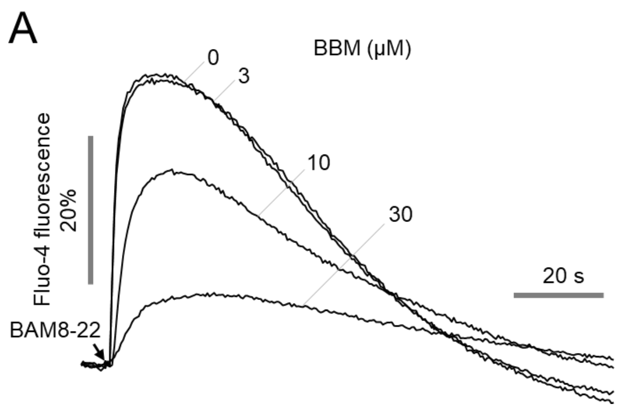

Figure S2. Effect of berbamine on BAM8-22 induced MrgprX1 activation. (**A**) Intracellular calcium levels were measured using Fluo-4 NW in HEK293T cells expressing MrgprX1. The cells were pretreated with indicated concentrations of berbamine (BBM) for 10 min prior to the treatment of 500 nM BAM8-22.

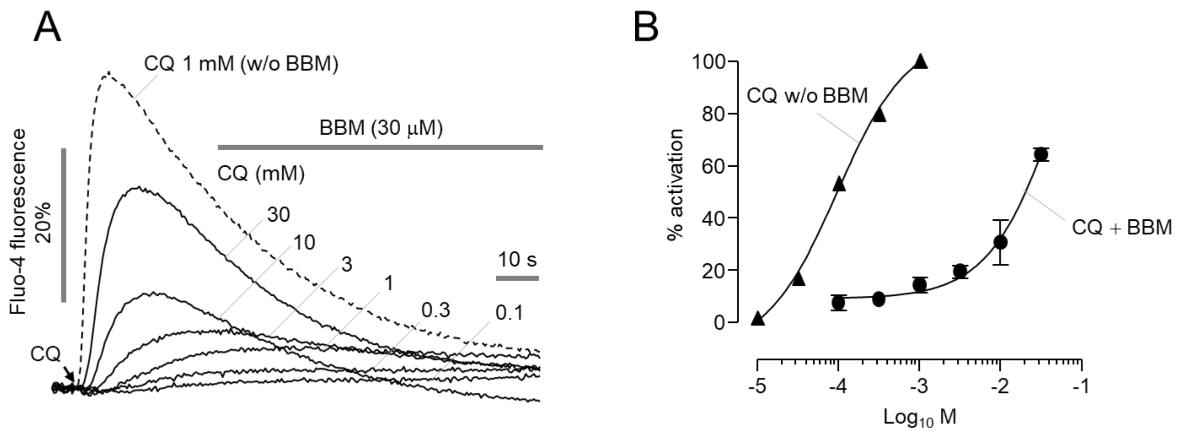

Figure S3. Dose response of CQ-induced MrgprX1 activation in the presence or absence of berbamine. (**A**) Intracellular calcium levels were measured using Fluo-4 NW in HEK293T cells expressing MrgprX1. The cells were pretreated with 30 μM of berbamine (BBM) and applied with indicated concentrations of CQ. (**B**) Summary of dose-response (mean ± S.E., n = 4). The dose response data of CQ in the absence of BBM was obtained from Figure 1B.
